# Prevalence and correlates of intimate partner violence against women: results from Mozambique Demographic and Health Survey 2022–2023

**DOI:** 10.3389/fpubh.2025.1568324

**Published:** 2025-07-21

**Authors:** Masood Ali Shaikh

**Affiliations:** Department of Medicine, College of Medicine, Korea University, Seoul, Republic of Korea

**Keywords:** intimate partner violence, alcohol, disparity, women, Mozambique

## Abstract

**Background:**

Intimate partner violence (IPV) is a widespread public health problem that affects women worldwide and represents a significant violation of human rights.

**Methods:**

This study utilized deidentified data from the 2022–2023 Mozambique Demographic and Health Survey to conduct a secondary analysis on the lifetime prevalence of IPV perpetrated by their current or most recent husband/intimate partner, and its associated factors. The analysis focused on 12 experiential, attitudinal, and socio-demographic attributes in women aged 15–49. For bivariate analysis, simple binary logistic regression models were used to identify correlates at the cutoff level of ≤0.20 *p*-value, which were then incorporated into the multivariable binary logistic regression model to analyze their associations with IPV. Model fit and collinearity were assessed to determine the utility of the multivariable analysis.

**Results:**

Altogether, 32.65% of women surveyed reported having experienced emotional, physical, or sexual violence from their current or most recent husband (for ever-married women) or intimate partner (for never-married women). Emotional IPV was the most common form, affecting 22.40% of respondents, closely followed by physical IPV at 21.34%. Women’s involvement in decision-making, their partner’s use of alcohol and controlling behavior, and knowledge of their father having ever beaten their mother were found to be statistically significantly associated with IPV in the multivariable model.

**Conclusion:**

IPV is shaped by a multitude of complex factors. One out of three women reported having experienced IPV, stressing the need for effective public health and societal measures to address and eradicate IPV in Mozambique.

## Introduction

Intimate Partner Violence (IPV) against women, perpetrated by their male partners is a global public health and human rights challenge (1). IPV involves physical, sexual, and/or emotional violence presenting in romantic relationships. With adverse implications for women’s physical and mental well-being, in addition to economic and social costs on communities (1).

The World Health Organization (WHO) defines IPV as “behavior within an intimate relationship that causes physical, sexual or psychological harm, including acts of physical aggression, sexual coercion, psychological abuse and controlling behaviors” (2). And estimates that globally, about one in four women (27%) aged 15 to 49, have experienced physical or sexual violence in their lifetime, perpetrated by their intimate partner (1).

In the WHO’s African region (WHO AFRO), the lifetime prevalence of physical and/or sexual IPV perpetrated by current or former husband or male intimate partner among ever-married or partnered women was estimated to be 33% in 2018 (3).

Mozambique, a country in southeastern Africa with a history replete with armed conflict, economic instability, and natural disasters, all of which contribute to a context where gender-based violence (GBV), including IPV, may thrive. Despite legal reforms and the introduction of national policies aimed at combating IPV (4, 5), the phenomenon remains deeply entrenched, often exacerbated by factors such as poverty, low levels of education, and cultural practices that endorse male dominance and control over women.

Previous studies and surveys, have highlighted the alarming rates of IPV in Mozambique. The 2011 Demographic and Health Survey data revealed that a significant proportion (39.6%) of married or in-union women aged 15–49 in Mozambique had experienced some form of IPV in the past 12 months, either frequently or sometimes (6). A 6-month prospective hospital-based study, conducted in 2011 in Beria city in Mozambique, reported 28% incidence rate of domestic violence in women (7). A study conducted in the secondary school attending women aged 15–25 in Maputo, Mozambique, with 413 participants providing information on their IPV experiences, 248 (60%) reported lifetime experience of at least one IPV type (8). While another single hospital-based study in Beria, Mozambique reported 1,491 admissions of domestic violence over the course of 5 years from 2011 to 2015; out of which 1,307 were among females, with 90% of them reported perpetrator as a current or former intimate partner (9).

The United Nations Sustainable Development Goal (SDG) 5, target number 5.2 has the noble goal to ‘Eliminate all forms of violence against all women and girls in the public and private spheres, including trafficking and sexual and other types of exploitation’, by 2030 (10). To track its attainment, it is imperative to study national-level IPV estimates and their correlates. However, there is dearth of such estimates in Mozambique. The Demographic and Health Surveys (DHS) are managed by the U.S. Agency for International Development (USAID) through its specialized Demographic and Health Surveys Program. These surveys offer nationally and sub-nationally representative data for numerous developing nations, delivering dependable and thorough metrics on IPV. Utilizing a standardized approach, DHS surveys have been conducted in more than 90 countries, including Mozambique (11).

The extensive data from these surveys illuminate the complex relationships between intimate partner violence (IPV) and various factors. Research utilizing DHS and other data sources highlights connections between IPV and significant aspects of women’s lives, such as age, educational attainment of both women and their partners, employment status, family wealth, controlling behaviors by male partners, alcohol consumption by male partners, and women’s experiences of violence during childhood and their attitudes toward IPV (12–44). Nevertheless, these reported results are far from uniform and consistent across countries, highlighting the variances and providing the imperative impetus for examining IPV in each country to gain a deeper understanding of this global public health and women’s issue. For instance, studies on the association between IPV and household wealth, residential status, and women’s age have reported mixed results that reflect the complex associations specific to countries. Findings from this study show the patina of these associations with IPV specific to Mozambique.

The most recent DHS in Mozambique was completed in 2022–2023, marking the fourth DHS in the country. However, only the 2011 and 2022–2023 DHS collected information on IPV. Therefore, the Mozambique DHS 2022–2023 provides the most recent national and subnational IPV indices.

Although there is increasing awareness of the need to tackle IPV in Mozambique, substantial research gaps remain. The scarcity of nationally representative data and methodological difficulties impede accurate assessment of IPV’s prevalence and dynamics. Currently, there are no nationally representative studies on IPV in Mozambique, except for the DHS 2011. This study sought to address this gap and enhance the limited understanding of IPV in Mozambique by presenting the prevalence and associated factors of IPV in the country. It utilized deidentified data from the latest nationally representative cross-sectional survey for secondary analysis. The study aimed to illuminate the intricate cultural, social, and economic dynamics that influence IPV in Mozambique.

## Methods

### Study area and data source

The Demographic and Health Surveys (DHS) employ a standardized methodology for conducting their surveys. The 2022–2023 Mozambique Demographic and Health Survey (MDHS2022-23) was implemented by the National Institute of Statistics (INE) in collaboration with Ministry of Health (MISAU) and the Institute National Health (INS). While the technical support was provided by the ICF International Inc. through the DHS Program. The data collection phase of this fourth DHS in the country lasted from July 27, 2022 to the 27th of February 2023. The sample design for MDHS2022-23 was a two-step process, aiming to provide estimates for the national level, urban and rural areas and each of the provinces, and for the capital Maputo City. The first step involved the selection of sample clusters, consisting of Enumeration Areas (EA), based on based on the fourth population census conducted in 2017. In total 619 EAs were selected with probability proportional to size. The second stage entailed 26 households were systematically selected in each EA with equal probability. All women aged 15–49 years in the household were eligible to be interviewed, and were included in the MDHS2022-23. The domestic violence (DV) module, which included IPV questions, was administered after obtaining verbal informed consent^1^, following the WHO guidelines on the ethical conduct of research on DV in women (45). In total, 4,813 women successfully completed the DV module. The IPV questions were asked from women who were having or have had intimate relationship, and defined as: *“Intimate partner: a man with whom a never-married woman is in a relationship that involves physical and/or emotional intimacy and for which the relationship is or has the expectation of being longer lasting,”* and a *“Husband/intimate partner: the current husband for currently married women; the most recent husband for divorced, separated, or widowed women; the current intimate partner for never-married women who currently have an intimate partner; and the most recent intimate partner for never-married women who do not currently have an intimate partner but had one in the past.”* While a boyfriend was not considered as an intimate partner if respondent defined him as “*a man with whom a woman has a causal relationship and who she did not mention as an intimate partner*.”

Details on the MDHS2022-23 sampling design, methodology, questionnaires, and survey implementation plan are provided in the Mozambique DHS country report, which is available for free download as a PDF document on the DHS program website (46). The results presented in this study are derived from a secondary analysis of fully deidentified data from the 2022–2023 Mozambique Demographic and Health Survey. This dataset was accessed after a successful application and approval for secondary analysis from the Measure DHS program^2^. All DHS datasets are made available to researchers worldwide for secondary analysis by the DHS program. Access is granted upon submission and approval of a brief description outlining the intended use for secondary analysis.

The Mozambique National Committee of Bioethics for Health (CNBS) granted the ethical clearance for the MDHS2022-23, in addition to the ICF International Inc. Institutional Review Board; ensuring that the survey procedures adhered to Mozambique’s ethical research standards, as well as those of the United States and international ethical guidelines.

### Study variables

The MDHS2022-23 included a domestic violence module based on a modified Conflict Tactics Scale (CTS), a widely recognized and reliable tool used in various settings (47, 48). This module features specific questions designed to assess emotional, physical, and sexual violence inflicted by male intimate partners. Additional details on how the outcome and explanatory variables were computed from this data are provided below.

### Outcome variable

Composite binary variable IPV was created and coded affirmative if the respondent reported having ever experienced any type of either emotional, physical, and/or sexual violence perpetrated by either her current or most recent male husband/partner. Emotional violence involved having ever being felt belittled, insulted, humiliated, or threatened with harm; physical violence involved having experienced being physically shaken, pushed, dragged, slapped, kicked, punched, arm twisted, hair pulled, any object thrown at, dragged, burned, or threated with any weapon like gun or knife; sexual violence involved having been coerced into performance of unwanted sexual acts, physically forced into unwanted sexual acts, or unwanted sex.

### Explanatory variables

Numerous studies have identified factors associated with IPV using data from the Demographic and Health Surveys (DHS), as well as from other sources. In this secondary analysis of deidentified DHS data, 12 potential associations were examined, including socioeconomic and demographic characteristics, women’s empowerment and acceptance of IPV, women’s awareness of IPV at home, the intimate partner’s alcohol use, and his controlling behavior. Socioeconomic factors considered were household wealth quintile, rural or urban residency, and the number of living children.

Women’s empowerment was assessed based on their ability to participate in decision-making regarding their own healthcare, visiting friends or family, and making major purchases, either independently or with their intimate partner. Attitudes toward IPV were evaluated by determining whether respondents justified wife beating in specific situations, such as refusing sex, arguing, neglecting children, burning food, or leaving the house without informing their husband. Exposure to violence was assessed by asking respondents if they knowledge about their father had ever physically assaulted their mother. The partner’s controlling behavior was evaluated by confirming experiences such as jealousy or anger when the woman talked to other men, accusations of infidelity, restrictions on social interactions, prohibiting meetings with female friends, and demanding to know her constant whereabouts. Additionally, the study investigated the intimate partner’s alcohol consumption.

Inquiries about the educational levels of husbands or partners and women’s involvement in decision-making were specifically posed to women who were either currently married or currently living with a male intimate partner.

### Statistical analysis

Deidentified Mozambique dataset for the DHS2022-23 in STATA file format were acquired for secondary analysis from the DHS website. Descriptive analyses were conducted to summarize attributes and outcomes, entailing unweighted counts, the number of missing values, and weighted proportions calculated for all variables; missing values were not imputed. Since the outcome variable was binary, i.e., presence/absence of IPV, binary simple logistic regression models were then computed for each explanatory variable to determine their association with the binary IPV outcome variable. Variables with a *p*-value of ≤0.20 in the bivariable logistic regression were identified as candidates for inclusion in the multivariable logistic regression model. Multicollinearity between explanatory variables was evaluated using the Variance Inflation Factor (VIF), and the Goodness-of-Fit test was used to determine fit of the model. For all binary and the final multivariable model, odds ratios, their corresponding 95% confidence intervals, and levels of statistical significance, are reported. A *p*-value of less than 0.05 was deemed to indicate statistical significance. All design-based analyses were performed using STATA version 18 (StataCorp LLC, College Station, Texas, USA).

## Results

Out of 4,894 women selected for the ‘Domestic Violence’ module of the MDHS 2022–2023, 4,813 were interviewed. Due to privacy concerns, 81 women could not be interviewed. From the 4,813 women chosen and interviewed for the domestic violence module, 4,454 women who were either currently or previously in a union or cohabiting with a man in an intimate relationship were asked the IPV questions.

Alarmingly, 32.65% of the women surveyed reported experiencing emotional, physical, or sexual violence perpetrated by their current or most recent husband or intimate partner. The most common type of intimate partner violence was emotional (22.40%), closely followed by physical violence (21.34%).

Several women reported experiencing multiple forms of IPV. Specifically, 12.45% endured both physical and emotional abuse, 4.34% faced both physical and sexual violence, 3.99% encountered both emotional and sexual abuse, and 3.08% suffered from all three types of IPV. In total, 23.61% of women experienced physical and/or sexual violence from an intimate partner, highlighting the intricate landscape of IPV in Mozambique. The most commonly reported forms of IPV within the three types of IPV, i.e., sexual, emotional, and physical were respectively: ever being forced into unwanted sex (4.67%); ever been insulted or made to feel bad (17.20%); and ever been slapped (16.21%).

Among ever-partnered women, 25.41% (95% CI: 23.54–27.38) reported experiencing IPV perpetrated by their current or most recent husband or intimate partner in the 12 months preceding the survey. The highest prevalence was observed among women aged 30–34 years, with 29.47% (95% CI: 24.73–34.70) reporting recent IPV.

The unweighted (raw) counts and the weighted proportions of different forms of IPV, as well as all the covariates included in the analysis are shown in Table 1. The analysis incorporates 12 key factors, but data for “Husband/partner’s education” and “Decision making” were unavailable for 1,134 women. This gap in data arises because these particular questions were directed only at women who were either currently married or currently had an intimate male partner. As a result, the tables displaying statistical analysis for these variables are restricted to women who are presently married or living with a man.

**Table 1:** Counts and proportions of study variables – Mozambique Demographic and Health Survey 2022–2023.

Variable	Unweighted count (*N* = 4,454)	Percentage (Weighted)
Outcome variable		
Intimate partner violence (Emotional, physical, and/or sexual)	Yes = 1,448	32.65 (30.63–34.74)
Emotional violence	Yes = 1,003	22.40 (20.68–24.22)
Physical violence	Yes = 960	21.34 (19.54–23.25)
Sexual violence	Yes = 334	6.62 (5.74–7.63)
Explanatory variables		
Age	15-19y = 585	13.48%
	20-24y = 928	22.24%
	25-29y = 846	18.74%
	30-34y = 642	12.70%
	35-39y = 611	13.12%
	40-44y = 461	10.36%
	45-49y = 381	9.35%
Respondent’s Education	No education = 1,189	29.56%
	Primary = 1,929	42.47%
	Secondary = 1,185	25.03%
	Higher = 151	2.94%
Husband/Partner’s education	No education/Do not know = 1,025	34.62%
	Primary = 1,306	37.87%
	Secondary = 856	24.04%
	Higher = 133	3.47%
	*Not Applicable = 1,134	
Occupation	Professional/Technical/Managerial/Clerical/	
	Sales = 813	15.69%
	Not working = 2,522	63.95%
	Agriculture: self-employed/	
	Services/Skilled manual/	
	Unskilled manual = 1,092	20.36%
	Missing = 27	
Wealth	Poorest = 733	19.26%
	Poorer = 720	17.93%
	Middle = 889	19.44%
	Richer = 994	21.25%
	Richest = 1,118	22.12%
Residence	Urban = 1,749	37.27%
	Rural = 2,705	62.73%
Children	0 = 647	15.26%
	1–2 = 1,669	37.06%
	3–4 = 1,268	27.65%
	5–12 = 870	20.03%
Decision making	Participated = 2,683	76.79%
	Not participated = 637	23.21%
	*Not Applicable = 1,134	
Acceptance	Not justified/Do not know = 3,655	80.23%
	Justified = 799	19.77%
Alcohol use by partner/husband	No = 2,978	71.76%
	Yes = 1,476	28.24%
Knowledge of Parental IPV	No/Do not know = 3,769	85.05%
	Yes = 685	14.95%
Controlling behavior	No = 2,202	50.41%
	Yes = 2,252	49.59%

* Questions on ‘Husband/partner’s education’ and questions pertaining to ‘Decision making’ were asked from only those women who were either currently married or currently living with a male intimate partner.

The respondent characteristics revealed that 54.46% women were aged 15 to 29 years while the rest were aged between 30 to 49; 67.50% women had achieved primary or secondary education; 61.91% of women reported analogous educational levels for their husband/partner. About two-quarter (63.95%) did not work. Respondents in poorest and poorer wealth quantile comprised of 37.19%; rural residence was reported by 62.73% respondents. Roughly half (47.68%) reported having three to 12 living children. Involvement in decision-making was reported by 76.79%; while acceptance of IPV in terms of justifying it, was reported by 19.77%. Intimate partner’s alcohol use was reported by 28.24% women; knowledge of father having beaten mother was reported by 14.95%; while half (49.59%) of women reported controlling behavior on the part of their intimate partner.

Results of bivariate and multivariable analysis obtained from simple and multivariable logistic regression models are reported in Table 2. Crude and adjusted odds ratios along with their corresponding 95% confidence intervals and significance levels are displayed. Of the 12 correlates assessed, 10 satisfied the selection criteria and were included in the multivariable model, while wealth and residential status did not meet the threshold for inclusion. Five of the ten correlates of IPV were not found to be statistically significantly associated with the IPV in the multivariable logistic regression model. These correlates included the number of children, the woman’s age, level of educational attainment, employment status, and attitudinal acceptance of IPV.

**Table 2:** Crude odds ratios, and adjusted odds ratios for all statistically significant associations between intimate partner violence and the selected variables – Mozambique Demographic and Health Survey 2022–2023.

Explanatory variable	Unadjusted OR	*p*-value	95% CI	Adjusted OR	*p*-value	95% CI
Age						
15–19	Reference		Reference			
20–24	1.40	0.038	1.02–1.93	1.10	0.644	0.74–1.63
25–29	1.47	0.011	1.09–1.99	1.09	0.681	0.71–1.68
30–34	1.81	<0.001	1.30–2.51	1.31	0.254	0.82–2.08
35–39	1.58	0.004	1.16–2.15	1.24	0.389	0.76–2.01
40–44	1.46	0.060	0.98–2.15	0.90	0.679	0.54–1.49
45–49	1.23	0.324	0.82–1.84	0.79	0.445	0.42–1.46
Education (Respondent/Women)						
No education	Reference		Reference			
Primary	1.15	0.192	0.93–1.43	1.10	0.452	0.85–1.43
Secondary	0.93	0.543	0.72–1.19	0.75	0.132	0.51–1.09
Higher	0.63	0.089	0.37–1.07	0.66	0.332	0.29–1.53
Education (husband/partner)						
No education	Reference		Reference			
Primary	0.95	0.678	0.76–1.20	0.77	0.043	0.60–0.99
Secondary	1.04	0.787	0.80–1.34	0.92	0.616	0.68–1.26
Higher	0.67	0.117	0.40–1.11	0.55	0.084	0.27–1.09
Occupation						
Professional technical, managerial, clerical, sales	Reference		Reference			
Not working	0.75	0.007	0.61–0.92	0.74	0.068	0.53–1.02
Agriculture: self-employed or employee/Household & Domestic/Services/ Skilled manual/Unskilled manual	1.16	0.197	0.92–1.46	1.05	0.782	0.73–1.52
Wealth						
Poorest	Reference			Not Applicable		
Poorer	1.00	0.985	0.67–1.51			
Middle	0.96	0.796	0.73–1.28			
Richer	1.07	0.635	0.80–1.43			
Richest	0.99	0.939	0.75–1.30			
Residence						
Urban	Reference			Not applicable		
Rural	1.01	0.907	0.84–1.22			
Children						
No children	Reference			Reference		
1–2 children	1.24	0.120	0.94–1.64	0.93	0.652	0.66–1.29
3–4 children	1.28	0.097	0.96–1.70	0.95	0.791	0.64–1.41
5–12 children	1.29	0.090	0.96–1.74	1.08	0.728	0.70–1.67
Decision making						
Did not participate	Reference			Reference		
Participated	0.81	0.127	0.62–1.06	0.62	0.002	0.46–0.84
Acceptance of IPV						
Not justified		Reference	Reference			
Justified	1.27	0.048	1.001–1.62	1.13	0.392	0.86–1.48
Alcohol use by partner/husband						
Does not use alcohol	Reference	Reference				
Uses alcohol	2.37	<0.001	1.96–2.87	1.88	<0.001	1.49–2.38
Knowledge of Parental IPV						
No	Reference		Reference			
Yes	2.31	<0.001	1.83–2.91	2.03	<0.001	1.53–2.68
Controlling behavior						
No	Reference		Reference			
Yes	5.98	<0.001	4.84–7.39	5.44	<0.001	4.37–6.76

OR, Odds Ratio; CI, Confidence Interval.

Table 2 also shows the multivariable logistic regression model results. Women whose husbands or intimate partners had attained primary education were significantly less likely to report experiencing IPV compared to those whose partners had no formal education (aOR: 0.77; 95% CI: 0.60–0.99), suggesting a protective association. Similarly, women who were involved in household decision-making had a lower likelihood of reporting IPV compared to those who did not participate in decision-making (aOR: 0.62; 95% CI: 0.46–0.84). Alcohol use by husband/partner was statistically highly significantly associated with higher odds of IPV reporting by women (aOR: 1.88; 95% CI: 1.49–2.38). Knowing about father having ever beaten mother was also statistically highly significantly associated with IPV reporting in women (aOR: 2.03; 95% CI: 1.53–2.68). Similarly, women reporting controlling behavior on the part of their husband/partner had higher odds of IPV (aOR: 5.44; 95% CI: 4.37–6.76). While association between women accepting of IPV and reporting IPV was not statistically significant. Since the questions on women’s involvement in decision-making and their husband or partner’s educational attainment were only asked of women who were currently married or had an intimate partner, the multivariable logistic regression model was restricted to this subgroup. Accordingly, all results pertain exclusively to women in a current marital or intimate relationship.

VIF results were less than 1.79 for all the covariates; the results of the goodness-of-fit test indicated that multivariable logistic regression model for the IPV was a good fit [*F*(9, 580)] = 0.80; *p*-value: 0.6141.

## Discussion

This study used the most recent nationally representative data on the prevalence and correlates of various types of IPV in Mozambique. The Demographic and Health Surveys (DHS) have been carried out in over 90 countries worldwide using standardized methods and tools that enable internationally comparable IPV estimates. The findings from the Mozambique Demographic and Health Survey of 2022–2023 (MDHS2022-23) reveal that about 1 in 3 women (32.65%) have experienced some form of IPV from their current or most recent husband or male intimate partner. Additionally, 23.61% of women reported physical and/or sexual IPV perpetrated by their current or most recent husband or male intimate partner. These figures contrast with the WHO’s AFRO region prevalence of 33% and the global estimate of 27% for these two types of IPV (3).

The most common type of IPV reported was emotional, closely followed by physical IPV. The difference in percentage points between the two was 1.06. Emotional IPV has been previously reported to be the commonest form (12, 14), while in other countries physical IPV was found to be the commonest type (13, 15, 16, 22).

Based on simple binary logistic regression models examining the individual associations between IPV and each of the 12 covariates, wealth and place of residence (urban vs. rural) did not meet the predefined threshold for inclusion in the multivariable model. Furthermore, variables such as the woman’s age, level of education, occupational status, number of living children, and acceptance of IPV were not found to be statistically significantly associated with IPV in the multivariable logistic regression analysis.

Many studies have shown that higher household wealth and economic status can shield against IPV (18, 19, 22, 32, 40). Conversely, increased IPV rates have been associated with lower wealth indices (26). However, some research has found no significant link between wealth and IPV (12–15). The link between residential status (urban vs. rural) and IPV is multifaceted, with research yielding mixed findings. Some studies indicate higher IPV rates in rural areas (25, 32), while others report lower rates (17). Additionally, some research has found no significant association between residential status and IPV (12–15, 22).

Similarly, studies on the association between women’s age and IPV has produced mixed results too. Some studies suggest that older women are at a higher risk of experiencing IPV (13, 16), while others suggest that older age may provide some level of protection against IPV (18, 39). On the other hand, younger age of women has been associated with a higher likelihood of IPV (21), yet several studies also report no statistically significant relationship between the age of women and IPV (12, 14, 15, 22). These differing findings highlight the complex socio-cultural influences that underpin the association between women’s’ age and IPV. Potential explanations entail older women having spent more time in intimate relationships, thereby increasing their risk and exposure to IPV. While younger couples might be lacking the skills and experience required to more effectively address and manage the stresses and challenges that involve intimate relationships.

Research suggests that a woman’s income and employment status may serve as protective factors against IPV (35, 36). However, when a woman earns more than her intimate partner, this dynamic can increase her risk of experiencing IPV (19, 36). Furthermore, numerous studies have found no clear association between occupational status and IPV (12–15, 22). Economic independence from a husband or intimate partner may reduce the traditionally submissive role expected of women in society. However, when a woman earns more than her husband or partner, it can threaten the male ego in some cases, leading to violence as a means of reasserting control and dominance.

Previous research has quite consistently identified having more children as a factor linked to higher IPV rates when compared to women without children (12–15, 17, 21, 32, 35). Though, one study found no significant link between the number of children and IPV (22), and another observed increased IPV rates among infertile women (36). These findings highlight that, in patriarchal societies, men may use violence when they feel inadequate in fulfilling the traditional role of provider. Similarly, infertile women may be subjected to violence due to societal pressures and expectations regarding their role in childbearing.

Research on women’s acceptance of IPV has produced mixed results; some studies find an association between acceptance and experiencing IPV (13–17, 38), while others report no such link (12, 22). Acceptance of IPV among women is often influenced by deeply ingrained cultural norms, societal practices, and personal upbringing. This learned behavior can reinforce a cycle of abuse within intimate relationships, making it difficult to break the pattern of violence. However, in the multivariable model, this correlate was not found to be statistically significantly associated with IPV. Suggesting that the five statistically significant correlates in the model may exert a stronger influence. Nevertheless, over 82% respondents stated that IPV Is not justified. Perhaps diminishing its value in deciphering association with IPV.

Education fosters understanding and promotes the use of peaceful approaches to resolving conflicts and overcoming life’s challenges. Several studies have assessed the association between IPV and the educational attainment of women and their partners, but they report inconsistent relationships. Studies have shown that women with lower educational levels are more likely to experience higher rates of IPV (21, 25), whereas having primary or higher education is generally associated with reduced IPV risk (14, 16, 19, 22). However, other research has reported no such relationship between education levels and IPV (12, 13, 15). Research indicates that women are more likely to report higher IPV rates when their male partners have low educational attainment (21, 24, 35), while partners with primary or higher education levels tend to be associated with lower IPV rates (14, 16, 19, 22). However, one study found that women with partners who had secondary education faced higher IPV compared to those whose partners were uneducated (13). Some studies also report no clear link between a partner’s education level and IPV (12). It is important to note that sociocultural factors can sometimes diminish the protective effects of education, influencing women’s rights and safety within intimate relationships.

The nuanced variations across countries highlight that ultimately “all epidemiology is local” (49). Understanding these differences is crucial for tackling and ultimately eradicating IPV, in line with the United Nations Sustainable Development Goals. The multivariable model findings for five IPV correlates that were found to be statistically significantly associated with IPV in Mozambique, reveal a complex and diverse array of IPV victim profiles. However, each individual correlate may affect individually and interact in consort with other correlates, resulting in association with IPV.

Several studies have reported no association between IPV and decision-making involvement (12–15, 22), while one study reported that it bestows protection from IPV (24). The connection between high levels of IPV and an intimate partner’s alcohol consumption is extensively documented (12–15, 17, 18, 20, 21, 24, 29, 35). Numerous studies have shown that alcohol consumption is a significant correlate of IPV. This relationship is primarily due to alcohol’s effects on the brain, which can reduce inhibitions and impair judgment, leading to an increased likelihood of violent behavior.

Knowing about father having perpetrated physical violence against mother is a painful experience and has been consistently associated with higher levels of IPV in women in their own intimate relationships (12–14, 17, 18, 22, 23, 28, 34). This association underscores the potential long-term impact of tolerance, acceptance and normalizing such violence in the context of intimacy.

The association between IPV and controlling behavior by male partner is another well-documented correlate (12–14, 17, 18, 22, 30). Controlling behaviors, such as isolating a partner from friends and family, and monitoring their activities can create an environment of fear and dependency. This environment can serve as a precursor to physical violence, as the controlling partner seeks to maintain power and dominance. Controlling behavior can escalate into IPV for several reasons. Firstly, it establishes a dynamic where the controlling partner feels entitled to dictate the terms of the relationship, including the use of violence to enforce compliance. Secondly, the victim’s isolation and dependency make it harder for them to seek help or leave the relationship, increasing their vulnerability to further abuse. The controlling partner’s behavior can also erode the victim’s support network, leaving them feeling trapped and powerless.

The results based on the secondary analysis of the MDHS 2022–2023 have practical implications for addressing IPV against women by their current or most recent husbands/partners. One-third of women reported such experience that is substantially high, with its attendant implications for health and human rights. The statistically highly significant associations of IPV with partners’ controlling behaviors and alcohol use, together with having knowledge of physical IPV perpetrated by respondents’ father on their mother suggest that there is no quick fix for this serious health and human rights concern in the country. Changing cultural norms and mores by educating men – and boys – about fundamental unfairness in controlling one’s intimate partner, will require long-term investments in health education and promotion. Such deeply engrained attitudes on controlling and beating one’s wife – as observed my daughters at home – will be an inter-and-multiple-generational effort. Similarly, deleterious alcohol effects on judgment need to be better understood by men to stem the tide of IPV. Albeit IPV is a part of a wider spectrum of violence, but this study used WHO’s IPV definition, with very clear and specific examples of what it entails, against the backdrop of power dynamics within intimate relationships.

The major strengths of this study are the use of the most recent and nationally representative data to understand the correlates of IPV in Mozambique. Every study has its limitations, and the primary limitation of this study involves the reporting of IPV perpetrated by the current husband for currently married women or the most recent husband for divorced, separated, or widowed women. For never-married women, the IPV questions pertained to the current or most recent intimate partner. Secondly, the self-reported nature of data collection introduces social desirability bias. Self-reported data can lead to underestimation of IPV, as feelings of shame and social stigma may prevent women from providing accurate disclosures.

Thirdly, the exclusive focus of IPV questions was on heterosexual relationships. Fourthly, women aged 15 to 49 years were included in MDHS 2022–2023. Women do not necessarily stop experiencing intimate partner violence once they reach the age of 50. Together these limitations underscore the underreporting of the true burden of IPV in Mozambique. Other limitations include the cross-sectional study design, which only allows for the determination of correlations rather than causation. There is also the possibility of reverse causation bias, where previous experiences of IPV might influence one’s attitudes toward IPV or alter memories of parental violence. Finally, because information on women’s participation in decision-making and their husband or partner’s educational attainment was collected only from women who were currently married or in an intimate partnership, the multivariable logistic regression analysis results are limited to this subgroup. As such, all reported results apply solely to women in a current marital or intimate relationship.

## Conclusion

Using the most recent nationally representative data on intimate partner violence against women, the lifetime prevalence of IPV perpetrated by the current or most recent husband/intimate partner, was 32.65% in Mozambique. This translates into one in three women having experienced IPV in their current intimate relationships. Controlling behavior and alcohol use by the intimate partner/husband, and knowledge of family violence, in terms of physical IPV committed by father on the mother were found to be statistically highly significantly associated with IPV. This high IPV burden in Mozambique underscores the imperative need for advancing gender equality, human rights, and improving women’s health. Hence, auguring the need for multifaceted responses tailored to Mozambique by health policymakers and practitioners using the most recent findings from the country.

## Data Availability

The data analyzed in this study is subject to the following licenses/restrictions: the Mozambique National Committee of Bioethics for Health (CNBS) granted the ethical clearance for the MDHS2022-23, in addition to the ICF International Inc. Institutional Review Board; ensuring that the survey procedures adhered to Mozambique’s ethical research standards, as well as those of the United States and international ethical guidelines. The approval for the conduct of secondary analysis of fully anonymized CDHS 2021-22 survey data was granted to the author by The Measure DHS using the online request form. Requests to access these datasets must be submitted to the DHS Program using the online portal available at Measure DHS website: (www.measuredhs.com).

## Footnotes

^1^ https://www.dhsprogram.com/Methodology/Protecting-the-Privacy-of-DHS-Survey-Respondents.cfm

^2^ www.measuredhs.com
